# Functional Roles of Matrix Metalloproteinases and Their Inhibitors in Melanoma

**DOI:** 10.3390/cells9051151

**Published:** 2020-05-07

**Authors:** Salvatore Napoli, Chiara Scuderi, Giuseppe Gattuso, Virginia Di Bella, Saverio Candido, Maria Sofia Basile, Massimo Libra, Luca Falzone

**Affiliations:** 1 Department of Biomedical and Biotechnological Sciences, University of Catania, 95123 Catania, Italy; napo.salvo@gmail.com (S.N.); scuderi331@gmail.com (C.S.); peppeg9305@gmail.com (G.G.); virgi.db95@hotmail.it (V.D.B.); scandido@unict.it (S.C.); sofiabasile@hotmail.it (M.S.B.); 2 Research Center for Prevention, Diagnosis and Treatment of Cancer, University of Catania, 95123 Catania, Italy; 3 Epidemiology Unit, IRCCS Istituto Nazionale Tumori “Fondazione G. Pascale”, 80131 Naples, Italy

**Keywords:** ECM, MMPs, melanoma, MMPi, MMP-9, OPN, TIMP, biomarker, therapy

## Abstract

The extracellular matrix (ECM) plays an important role in the regulation of the tissue microenvironment and in the maintenance of cellular homeostasis. Several proteins with a proteolytic activity toward several ECM components are involved in the regulation and remodeling of the ECM. Among these, Matrix Metalloproteinases (MMPs) are a class of peptidase able to remodel the ECM by favoring the tumor invasive processes. Of these peptidases, MMP-9 is the most involved in the development of cancer, including that of melanoma. Dysregulations of the MAPKs and PI3K/Akt signaling pathways can lead to an aberrant overexpression of *MMP-9*. Even ncRNAs are implicated in the aberrant production of MMP-9 protein, as well as other proteins responsible for the activation or inhibition of MMP-9, such as Osteopontin and Tissue Inhibitors of Metalloproteinases. Currently, there are different therapeutic approaches for melanoma, including targeted therapies and immunotherapies. However, no biomarkers are available for the prediction of the therapeutic response. In this context, several studies have tried to understand the diagnostic, prognostic and therapeutic potential of MMP-9 in melanoma patients by performing clinical trials with synthetic MMPs inhibitors. Therefore, MMP-9 may be considered a promising molecule for the management of melanoma patients due to its role as a biomarker and therapeutic target.

## 1. Introduction

The extracellular matrix (ECM) plays several roles in the regulation of cellular homeostasis and in the regulation of cell-cell interactions. ECM alteration leads to morphological and structural changes which are responsible for several pathological conditions [1]. Dysregulations in ECM structure and components are key events in tumor growth and cell migration favoring several processes, such as vascularization and loss of cell adhesion [2]. Indeed, the interaction between cells and ECM is mediated by several receptors and secretory molecules, capable not only of regulating connection and cell migration, but also in modulating the expression of the genes involved in cell growth and differentiation [3]. In this context, several studies have demonstrated that cell-ECM interactions regulate the molecular processes, which underlie cell differentiation [3], cellular homeostasis [4], wound healing [5], and cancer invasion [6,7].

ECM remodeling is finely regulated by several proteins involved in the cleavage and hydrolysis of the ECM component, favoring the pathological and physiological modification of ECM that have impacts on many cellular processes, as previously described [1,8].

Matrix Metalloproteinase (MMP) family is the protein family mainly associated with the degradation of the ECM. In humans, this family is composed of 24 proteins encoded by different genes and named MMP 1–28, although some MMPs are not human MMPs (MMP-4, MMP-5 and MMP-6) or are aliases of other MMPs (MMP-22). MMP family has numerous roles in physiology and fetal development, but it has been demonstrated that some of these proteinases, when dysregulated, can be precursor of pathological conditions [9].

MMP activities are strictly regulated by other proteins that ensure steady-state conditions between the degradative and reconstructive processes of the ECM [10]. In particular, MMPs are regulated by a class of endogenous inhibitors, named tissue inhibitors of metalloproteinase (TIMP), that are involved in both MMPs activation and inactivation [11]. The different structures characterizing the various MMPs allow the accomplishment of multiple functions influencing several processes like cell behavior, apoptosis and cell proliferation. Oncological studies have shown that MMPs play several functions favoring tumor progression through the degradation of surrounding tissues, the modulation of growth factors and membrane receptors, as well as inflammatory proteins, adhesion molecules and chemo-attractive proteins [12,13,14].

MMPs carry out an important role also in the modification of skin ECM. Indeed, MMPs are involved in skin matrix remodeling through the degradation and reconstruction of matrix components. Furthermore, several studies have demonstrated that MMPs play fundamental roles in melanoma, where tumor cells and tumor microenvironment alterations were associated with MMPs and TIMPs de-regulation [15].

Melanoma represents the most lethal tumor among all skin cancers because of its aggressiveness and its high metastatic potential. It represents about 3–5% of all skin cancers and it is often diagnosed in patients aged between 20–35 years or in patients with more than 65 years as a consequence of several risk factors, including chronic sunlight exposure, a clear phototype and specific somatic mutations [16,17,18]. Despite the evolution of cancer pharmacological treatments [19], several epidemiological studies have demonstrated that melanoma incidence is about 5%, while the mortality rate among all skin cancers is about 75%. In this regard, the last updated USA cancer statistics data shows that 20% of the population develops this tumor during their life [20,21].

Among the recognized risk factors for melanoma there are genetic susceptibility and gene polymorphisms [22]. Indeed, about 10–15% of melanomas are diagnosed in patients with a family history of melanoma. Another significant fraction of melanoma is associated with two different familial syndromes called familial atypical multiple mole melanoma syndrome (FAMMM syndrome) and the melanoma-astrocytoma syndrome (MAS) [23]. In addition, some germline mutations have been associated with a higher risk of melanoma. In particular, mutation occurring in the cyclin-dependent kinase inhibitor 2A (*CDKN2A*) and cyclin-dependent kinase 4 (*CDK4*) genes are the most frequent genetic abnormalities associated with melanoma risk [22,24].

As mentioned, besides these hereditary and familial risk factors, solar ultraviolet radiation (UV-A and UV-B rays) represents the most important trigger of melanocyte neoplastic transformation due to the induction of several genetic mutations [25,26,27].

According to Catalogue Of Somatic Mutations In Cancer (COSMIC), among these mutations the most frequently observed in melanoma are mutations affecting the *BRAF* (44%), *NRAS* (17%), *TERT* (25%), *CDKN2A* (18%), *KIT* (8%), *GRIN2A* (20%), *TP53* (15%), *PTPRT* (20%), *LRP1B* (26%), *NF1* (17%), and *PTEN* (10%) genes [28]. In particular, about 50% of melanoma is characterized by the presence of *BRAF* activating mutations. In 90% of cases, this mutation is represented by the *BRAF^V600E^* mutation where the valine 600 is substituted with the glutamic acid (p.V600E) that leads to the over-expression and hyperactivation of *BRAF* [29].

All these mutations are involved in the alteration of key molecular and signaling transduction pathways responsible for the abnormal proliferation of cancer cells and loss of apoptosis [30,31]. In addition, these somatic mutations, especially that of *BRAF*, are involved in the alteration of the tumor microenvironment through the regulation of the Mitogen-Activated Protein Kinase (MAPK) pathway. In particular, the hyperactivation of MAPKs pathways is associated with the over-expression of the transcription factor ERK that in turn leads to the over-expression of several genes involved in tumor development, including *MMPs*, *TGFβ*, and Osteopontin (*OPN*), whose role in melanoma is fundamental for the tumor invasion and metastatic processes [32,33,34].

Starting from these preliminary data on the physiological and pathological role of ECM and MMPs, and taking into account the epidemiological, molecular and mutational data of melanoma, this article aims to review the current knowledge on the involvement of MMPs. It will particularly focus on Matrix Metalloproteinase 9 (MMP-9), in the degradation of ECM and the consequent progression of melanoma, as well as the potential therapeutic implication of both endogenous and exogenous MMP inhibitors for the design of new therapeutic protocols for melanoma patients.

## 2. Matrix Metalloproteinases

The MMPs are a subgroup of metalloproteinases (including disintegrin, ADAM metalloproteinases and other proteolytic enzymes) containing a catalytic zinc ion site and an evolutionarily conserved methionine residue [35].

At present, 24 different MMPs were identified in humans and 23 in mice. All the MMPs are grouped in seven major categories according to their substrates or proteolytic functions: Collagenases, gelatinases, stromelysins, matrilysins, metalloelastase, membrane-type MMPs and other MMPs [36,37,38] (Table 1).

### 2.1. Collagenases

The group of MMPs with a collagenase activity contains three different MMPs: MMP-1, MMP-8, and MMP-13. MMP-1 is a collagenase found in the interstitial space able to cleave types I, II, and III collagens. Several studies showed that MMP-1 protein is over-expressed in several tumors and leads consequently to a more aggressive phenotype [39]. Although MMPs are generally considered as pro-oncogenic proteins, some MMPs, such as MMP-8, may have anti-tumor properties. Indeed, in different experimental models the neutrophil collagenase, MMP-8 showed anti-tumoral properties suppressing cell proliferation and the formation of metastases [12]. As described for MMP-1, also MMP-13 protein expression increased during the invasive processes of some tumors, including melanoma [41].

### 2.2. Gelatinases

This group contains MMP-2 and MMP-9, called also gelatinases A and B, respectively, with a gelatinolytic activity. In the following paragraphs, the function and regulation of MMP-9 will be widely described. Regarding MMP-2, it was demonstrated that MMP-2 protein is expressed in the stroma and cell cytoplasm. Furthermore, MMP-2 acts in the ECM by degrading the type I collagen, type IV collagen and other components, such as fibronectin and gelatin [42].

### 2.3. Stromelysins and Matrilysins

The group of stromelysin and matrilysin contains respectively MMP-3, MMP-10 and MMP-11 (Stromelysin) and MMP-7 and MMP-26 (Matrilysin). All these MMPs are involved in the degradation of different components of the ECM. In particular, stromelysins plays a major role in the degradation of proteoglycans, gelatin, and other constituents of the extracellular matrix [38], while the MMP-7 and MMP-26 matrilysins are associated with the cell membrane by binding to cholesterol sulphate and degrade substrates like fibronectin, laminin, collagen type IV, and gelatin. These MMPs are expressed in primary and metastatic melanoma [46].

### 2.4. Metalloelastase MMPs

The group of metalloelastase is only represented by MMP-12. This matrix metalloproteinase is able to hydrolyze soluble and insoluble elastin. It also degrades type IV collagen and fibronectin. It is involved in tissue remodeling after injuries [48]

### 2.5. Membrane-Type MMPs

Membrane-type matrix metalloproteinases (MT-MMPs) represents a subgroup of the MMP family composed of six MT-MMPs in humans [50,51]. There are two main groups of MT-MMPs: type I transmembrane-type (MT1-, MT2-, MT3- and MT5-MMPs) and glycosylphosphatidylinositol (GPI)-anchored type (MT4- and MT6-MMPs) [58]. All these MMPs are linked to the cell membrane, regulating key processes in the cell-cell and cell-ECM interactions [58]. The most investigated MT-MMPs is MT1-MMP (MMP-14). MMP-14 was the first discovered membrane-type MMP. This MMP is also involved in the invasive phenotype of some tumors, including melanoma [58], by interacting with MMP-2 in the stroma and promoting cancer progression [49]. In vivo, the cleaved form of laminin 332 was found in tumors and in tissues undergoing remodeling but not in quiescent tissues [38]. Cleavage fragments of the two chain of laminin 332 generated by MMP14 were detected in high invasive melanoma cells and played a crucial role in cell adhesion, migration and vasculogenic mimicry (VM) [59]. It was demonstrated also that BRAF and NRAS mutations positively regulate *MMP-14* gene expression enhancing tumor growth and melanoma invasiveness in vivo [60].

### 2.6. Other MMPs

Other MMPs, like MMP-19, MMP-21, MMP-23A and B, etc., are not clustered in a specific group, therefore are named “other MMPs”. Of these, the most studied are the MMP-19 and MMP-21 whose expression in melanoma cell lines was correlated, respectively, to a higher invasive power (MMP-19) and to the malignant transformation of melanocytes (MMP-21), suggesting a possible use of these MMPs as predictive biomarkers of cancer progression [52,54].

As shown, several studies have described the involvement of almost all of these MMPs in the development of cutaneous melanoma. It is clear that the strong involvement of MMPs in melanoma development and other skin cancers depends mainly on the specific features of melanoma that is a tumor characterized by a high invasive power toward the surrounding tissues and high rates of metastases and recurrence [61].

## 3. MMP-9 Functions and Regulation in Melanoma

MMP-9 is a protease involved in extracellular matrix degradation. In humans, the *MMP-9* gene is mapped in the genomic region 20q13.12 and codifies for a protein of 707 aa (92 kDa) secreted in the extracellular space as inactive pro-enzyme named pro-MMP-9. The pro-MMP-9 is inactive because of 80 aa residues at the N-terminal site where a cysteine switch motif coordinates the zinc ion forming the catalytic domain of the protein, thus maintaining it inactive [43,62].

**Figure 1:**
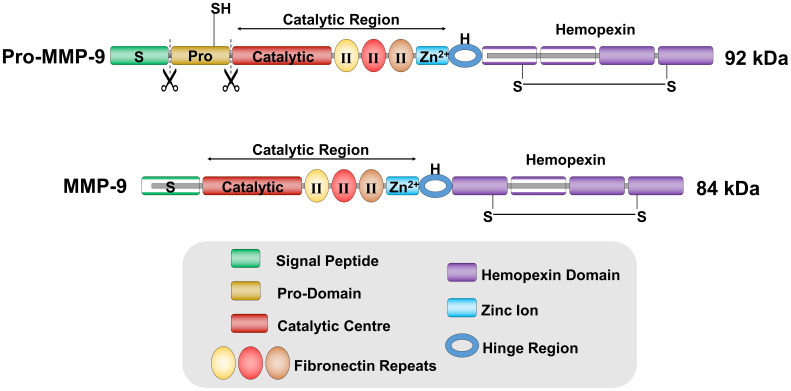
Protein structure of MMP-9. MMP-9 is first produced as a pro-enzyme of 92 kDa, called pro-MMP-9, containing a pro-domain of 73 aa responsible for MMP-9 catalytic latency. Subsequently, other proteases cleave pro-MMP-9 pro-domain generating the active catalytic form of MMP-9 of 84 kDa.

In the extracellular space other proteinases, like MMP-3 or MMP-2, cleave the inactive form of pro-MMP-9 in the active form of 84 kDa [63,64] (Figure 1).

MMP-9 is expressed in neutrophils, macrophages, and fibroblasts. Proangiogenic factors, including the fibroblast growth factor (FGF) and vascular endothelial growth factor (VEGF), are activated when MMP-9 degrades the ECM [65].

It was demonstrated that melanoma MMP-9 and MMP-2 play a fundamental role in the degradation of the ECM, thus, favoring melanoma spreading towards the surrounding tissues until the formation of distant metastases [16,34,66]. Several studies have demonstrated that the up-regulation of MMP-9 is strictly influenced by several genetic alterations or modifications of the tumor microenvironments [65,67,68]. In particular, it was proved that the high levels of *MMP-9* in melanoma patients might be due to the dysregulation of the TGFβ pathways where the alterations in the levels of NF-κB is able to induce the overexpression of *MMP-9* via OPN activation [34]. Moreover, it has also been demonstrated that epigenetic modifications may lead to the up-regulation of *MMP-9* in melanoma and other cancer types, as explained in the following paragraphs.

It was demonstrated that many molecular pathways, including Ras-Raf-MEK-ERK (MAPKs) and PI3K/PTEN/AKT/mTOR (PI3K/AKT), are associated with the regulation of *MMP-9*. Dysregulations in these pathways can lead to an aberrant over-expression of several inflammatory proteins and consequently to the increased expression of several ECM proteases, like MMP-9 [33].

The MAPKs pathway has a relevant role in the physiology of cells, initiating the phosphorylation cascade of different kinases proteins, yielding to cell growth and differentiation [69]. Activating mutations within this pathway can lead to the loss of negative feedback control, and in turn, to the over-activation of the pathway itself leading to uncontrolled cell proliferation [70]. For instance, *BRAF* mutations induce the constitutive activation of MAPKs pathway able to induce the hyperphosphorylation of the transcription factor ERK that in turn induce the up-regulation of genes involved in survival and proliferation of cancer cells [71]. Among these genes, there is also *MMP-9* whose degradative action towards ECM was already widely discussed [72,73].

Other mechanisms of MMP-9 over-expression in melanoma are mediated by neural crest associated genes, i.e., FOXD1, via the RAC1B pathway. Wu et al. (2018) have demonstrated that the up-regulation of *FOXD1* leads to the over-expression of MMP-9 mRNA and protein levels. In particular, the authors observed a decrease of MMP-9 expression by using a siRNA against *FOXD1*. In addition, the loss/down-regulation of *RAC1B* decreases the spreading of tumor cells and delays the Epithelial–Mesenchymal Transition, underlying the importance of the *FOXD1*-*RAC1B* axis in the regulation of MMP-9-mediated melanoma cells invasion [74].

Other studies have demonstrated that MMP-9 not only mediates the degradation of the ECM, but is also involved in neo-angiogenesis, cell migration and formation of metastases.

In this context, the most aggressive forms of melanoma are able to undergo VM, in which tumor cells behave like an endothelial cell forming microvascular channels, small interconnection in the ECM connecting different tissues, due to the action of proteinases and angiogenic factors [75]. Among these proteinases, MMP-2/9 showed both, in vitro and in vivo their implication in VM. In particular, the complex Rictor-mTORC2 is able to phosphorylate AKT, leading to the overexpression of MMP-2/9 and the formation of microvascular channels [76,77].

Finally, other regulatory mechanisms of *MMP-9* expression are mediated by *MMP-9* allelic variation (rs3918251GG (A>G) and by the over-expression of *SOX4* responsible for the concomitant up-regulation of *MMP-2/9* and *NF-κB*/*p65* and, consequently, for a more aggressive tumor phenotype [78,79].

## 4. Epigenetic Modulation of MMP-9 Expression

Besides the well-recognized genetic alterations associated with the up-regulation of *MMP-9*, recent evidence suggests that epigenetic events may alter the expression levels of this gene.

The development of new high-throughput technologies in the field of molecular biology allowed the collection of a huge amount of bioinformatics data regarding the expression profiles of both mRNA and non-coding RNA (ncRNA) [80,81,82].

**Figure 2:**
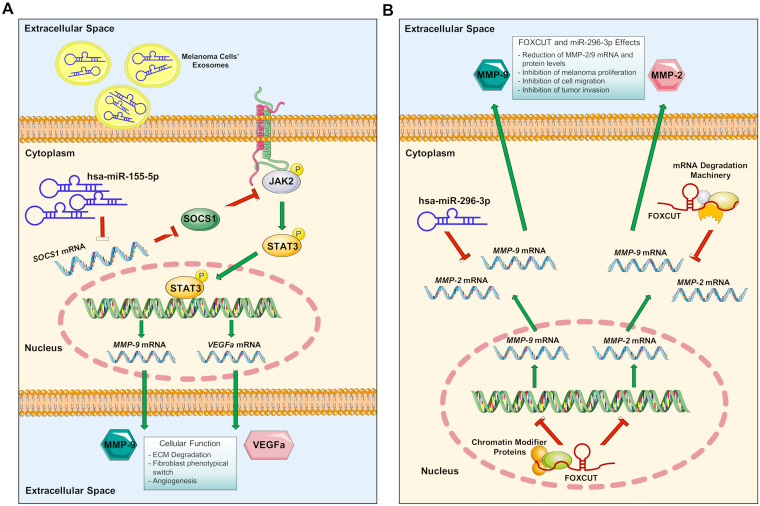
Epigenetic regulation of *MMP-9* in melanoma. (**A**) Melanoma cells secrete exosomes containing different proteins and ncRNAs, including hsa-miR-155-5p. These exosomes are internalized by cells of the tumor microenvironment, such as fibroblasts. Inside the cells the exosomal hsa-miR-155-5p is able to inhibit *SOCS1* mRNA thus activating the JAK/STAT molecular pathway. Phosphorylated STAT3 binds the DNA inducing the transcription of *MMP-9*, *VEGFa* and other pro-angiogenetic factors (e.g., *FGF2*). These proteins will be responsible for ECM degradation, fibroblasts switch to cancer-associated fibroblast phenotype and neoangiogenesis; (**B**) ncRNAs may act also as inhibitors of *MMP-9* and *MMP-2*. In particular, hsa-miR-296-3p is able to inhibit both *MMP-9* and *MMP-2* mRNA thus reducing their secretion in the extracellular space. Similarly, FOXCUT lncRNA is able to inhibit *MMP-2* and *MMP-9* transcription and translation into proteins. Both ncRNAs are responsible of the inhibition of melanoma cell proliferation, migration and invasion.

Through computational approaches, different studies have identified specific ncRNA associated with the development of cancer or with gene and protein alterations [66,83,84,85,86,87,88,89]. Specifically, through the analysis of bioinformatics data, it was possible to identify specific microRNAs (miRNAs) associated with melanoma or miRNAs able to selectively target and modulate *MMP-9* [66,84]. Some studies have tried to determine the role of ncRNAs in the regulation of MMP-9 (Figure 2).

Zhou et al. (2018) have shown high levels of miR-155-5p in exosomes derived from a co-culture of melanoma cells and fibroblasts. The authors have demonstrated also that miR-155-5p is able to inhibit *SOCS1* in fibroblast and in turn enhancing the expression levels of p-STAT3 responsible for *VEGFa* and *MMP-9* transcriptional activation. The epigenetic alterations of both *VEGFa* and *MMP-9* mediated by hsa-miR-155-5p were finally responsible for the phenotypical switch of fibroblasts towards a cancer-associated fibroblast phenotype known to be associated with angiogenesis [90].

In a study performed by Wang et al. (2018), two ncRNAs were computationally predicted to bind *MMP-9* and *MMP-2*. These ncRNAs are the FOXC promoter upstream transcript (FOXCUT) and the miRNA hsa-miR-296-3p. In particular, the authors have shown in C918 cells, a model for choroidal malignant melanoma (CMM, an interocular type of melanoma), that both MMP-2/9 transcripts and proteins were downregulated when FOXCUT and/or miR-296 were transfected in C918 cells [91].

## 5. Approved Therapeutic Approaches for Melanoma

The therapeutic approaches for the treatment of melanoma vary significantly depending on the pathological features of tumor. Most of the dysplastic nevi and non-invasive superficial melanoma are treated by surgical resection with wide skin margins free from disease. This type of surgical resection is conclusive in most cases. However, in cases of advanced-stage melanoma, surgical resection is often not applicable or must be accompanied by drug therapy [92].

The therapeutic efficacy of pharmacological anti-cancer treatment in melanoma depends on different factors, including the genetic landscape of cancer cells, tumor microenvironment alterations and epigenetic alterations. At present, the best therapeutic approach for melanoma patients consists in a combination of drugs that inhibits the kinase activity of BRAF (BRAFi) and MEK (MEKi), downstream effectors of the MAPK pathway; in addition, new treatments with immune checkpoint inhibitors are now being used for advanced or metastatic melanomas [19,93].

On the basis of the latest evidence, the combination of BRAFi and MEKi results in a better therapeutic response of melanoma patients positive to BRAF mutations [94]. Currently, the combination therapies available for melanoma are three: Vemurafenib and Cobimetinib, Dabrafenib and Trametinib, Encorafenib and Binimetinib. In particular, Dabrafenib is a RAF inhibitor used for patients with advanced or metastatic melanoma positive to the *BRAF^V600E^* mutation [95]. On the other hand, Trametinib is a MEK-1/2 inhibitor and it is also used for the treatment of advanced or metastatic melanoma with *BRAF^V600E^* mutation [96].

The identification of drugs capable of targeting key mutation involved in melanoma raised the attention on the identification of potential biomarkers of therapeutic response. In this context, several studies have proposed MMP-9 as a candidate marker used for the assessment of Dabrafenib therapeutic response. In particular, Salemi R et al. (2018) have analyzed the presence of circulating-free (cfDNA) *BRAF^V600E^* mutation in liquid biopsy samples obtained from patients treated with BRAFi and/or MEKi by using the droplet digital PCR system. The authors showed that the baseline presence of cfDNA *BRAF^V600E^* mutation was associated with a worse prognosis. Moreover, the authors have demonstrated that during the therapy there was a positive correlation between the number of cfDNA of *BRAF^V600E^* mutation and MMP-9 protein levels, thus proposing MMP-9 as a new biomarker of response or resistance to the treatment [32]. In this context, recent studies highlight how the analysis of circulating tumor DNA and liquid biopsy samples coupled with the use of high-sensitive genetic and proteomics techniques may represent innovative strategies to identify molecular biomarkers, including MMPs and related proteins, in order to early detect pre-cancerous lesion, tumor or tumor relapse [97,98].

As anticipated, in the last years, new drugs have been developed for the management of melanoma patients. These monoclonal antibodies are directed towards the PD-1, a surface protein expressed on cancer cells capable to inhibit the T-lymphocyte activation. These new FDA and EMA approved monoclonal antibodies are Pembrolizumab and Nivolumab [99,100].

Finally, in the past years, some studies have provided evidence that new combination therapies, with both selective inhibitors and immune checkpoint inhibitors, have a higher therapeutic efficacy compared to monotherapy [101]. Furthermore, several studies have proved that the concomitant administration of anticancer treatment and supportive care regimens with probiotics and antioxidant compounds is associated with a higher response rate compared to the treatments with only anticancer agents [102,103].

Overall, despite the different drugs available for the treatment of melanoma, a significant fraction of patients is refractory to therapies. Therefore, it is necessary to identify new effective treatments for the management of melanoma patients. In this context, the development of new selective inhibitors for MMPs could represent a promising strategy to improve the efficacy of current treatments and inhibits tumor aggressiveness.

## 6. Clinical Application of MMPs Inhibitors

Different molecules are able to interact with MMPs modulating their functions; these are called MMPs inhibitors (MMPi), which can be divided into synthetic inhibitors and endogenous inhibitors [104]. There are several inhibitory mechanisms of MMPs activity, but the most common involves the binding of the molecule with the zinc atom of the catalytic domain of the protein [105]. The compounds and functional groups used to inhibit the MMPs activity are summarized in Table 2. The first synthetic inhibitor was developed with a structure similar to that of collagen where the hydroxylated domain was recognized by the zinc atom of the catalytic site of MMPs, thus, acting as a competitive inhibitor of collagen [106,107]. Pharmaceutical companies began developing MMPi drugs since the 1990 for the treatment of pathologies, in which the proteinases are dysregulated, like in cancers. The first were Marimastat (BB-2516) and Cipemastat (Ro 32-3555), both containing the hydroxamate group [108].

Chirivi et al. (1994) have studied the efficacy of Batimastat, a first-generation MMPi, in C57BL/6N mice injected with B16-BL6 melanoma cells to evaluate the inhibition of tumor growth. The results showed a reduction of lung metastasis and solid tumor dimensions when the drug was administrated after the inoculation of cells [109]. Another in vivo study on a melanoma animal model was performed to assess the efficacy of Batimastat when it is administrated in combination with IL-12; also in this case, the results showed strong antitumoral and antiangiogenetic effects [110]. In a study conducted by Wylie et al. (1999), Batimastat induced anti-angiogenic effects after inoculation of B16F1 melanoma cells in mice, thus reducing sensibly the size of liver metastases without interfering with the extravasation of circulating melanoma cells [111].

Another MMPi with promising in vivo results was MMI270. This is a synthetic hydroxamic acid derivate, capable of inhibiting the degradative action of MMPs at nanomolar concentration. It did not show anti-proliferative activity in the primary tumor, but it was effective in reducing the angiogenic activity. Kasaoka et al. (2002) have studied how the administration of MMI270 can affect the extravasation of melanoma cells injected i.v. in a murine model. They have hypothesized the lymphatic system as the main route of metastasis formation, proposing the possibility of the administrating MMI270 after lymphadenectomy to reduce the metastatic niches number [112].

Conway et al., in 1996, have conducted a study on four synthetic MMPi in mice injected s.c. with melanoma cells. The drugs were pumped continuously through a s.c. implanted micropump to reach high plasmatic concentration. Despite the high concentration of drugs, the results demonstrated no significant difference in metastasis formation, thus excluding these four molecules from being used in human clinical trials [113].

Another study on B16-BL2 melanoma cells injected intramuscularly in C57B2/7 mice using a Recombinant Human Tissue Inhibitors of Metalloproteinases-2 (r-hTIMP-2) shown a significant decrease in metastasis formation, especially when the drug was administered right after the tumor injection [114].

MMI-166, a third-generation inhibitor of MMP-2 and MMP-9, has shown to be significantly effective in reducing tumor cell proliferation and the formation of metastasis. In 2002, Hojo and colleagues have studied this drug injecting B16-BL6 melanoma cells in mice. Their results have shown that MMI-116 is able to reduce tumor proliferation, and the combination with paclitaxel or carboplatin yield to an increase of antitumoral activity of the drug [115].

The promising results that came from preclinical studies using MMPis led to the development of clinical studies on humans. Batimastat (B394) had no success in human, because of its low bioavailability. Indeed, the results obtained from phase I clinical studies have revealed that prolonged treatment with MPIs can lead to toxic effects such as muscular-skeletal pain and inflammation [116].

Consequently, Agouron and Bayer have produced two new drugs Prinomastat (AG 3340) [117] and Tanomastat [118], a thiol-based inhibitor (BAY 12-9566), with the aim of minimizing, or completely eliminating, the adverse effects of the previous generation. The efficacy of these molecules is particularly direct toward MMP-9 and MMP-2. Unfortunately, Prinomastat (AG 3340) showed no efficacy during the administration of phase III trial on patients with Non-Small Cell Lung Cancer (NSCLC) and prostate cancer, and this caused the anticipation in the shutting down of the study [119]. For this reason, there are no data about progression-free survival (PFS) or overall survival (OS). Anyway, a good portion of patients was affected by muscular-skeletal toxicity. Moreover, the patients with NSCLC, who received chemotherapy in association with the MPI, had a greater chance of developing venous thromboembolism [120].

For Tanomastat, two clinical trials were done, without showing any reduction of tumor growth or reduction of PFS and OS. The first trial, a multicentric randomized double-blind compared to placebo study, was performed on advanced epithelial ovarian cancer [121]. The second trial was performed on pancreatic cancer patients to assess the efficacy of Tanomastat compared to that of gemcitabine. In this second study, the effect of gemcitabine was higher compared to those observed for Tanomastat [122].

Other clinical studies were performed to test the efficacy of Marimastat compared to gemcitabine. In particular, the trials performed in more than 400 patients with non-resectable pancreatic cancer showed that Marimastat did not show a higher therapeutic effect compared to Gemcitabine [123]. In another trial, Marimastat was compared to a placebo in 369 patients with unresectable gastric cancer, in which a moderate increase of the progression-free survival, but not in the overall survival, was observed [123].

Another study performed by administering BMS-275291 has shown exciting preliminary results during the first step of a clinical trial on NSCLS patients where lower dose-dependent toxic effects were observed. However, at the end of the trial it has been demonstrated that the concomitant administration of BMS-275291, Paclitaxel and Carboplatin did not confer any advantage for the improvement of OS, PFS and relative risk. Moreover, toxicity was too high when compared to the control group, showing a higher incidence of hypersensitivity reaction, febrile neutropenia and skin rash. Despite the promising preclinical results, the study was stopped earlier together with other two trials: One on patients with breast cancer, and another on prostate cancer [124].

**Table 2:** Structure of synthetic MMPi compounds.

Compound	Functional Group	Activity	Source
Marimastat	hydroxamate group (-CONHOH)	Binds to zinc domain of several MMPs	[108]
Prinomastat	hydroxamate group (-CONHOH) and Aryl backbone	Selectively inhibits MMP-2, 9, 13, and 14	[117]
Rebimastat	Thiol	Selectively inhibits MMP-1, 2, 8, 9, and 14	[118]
Tanomastat	Thiol	Binds to zinc and selectively inhibits of MMP-2, 3, and 9	[118]
Ro 28-2653	Pyrimidine	Selectively inhibitsMMP-2, MMP-9 and membrane type 1-MMP	[125]
3-hydroxypyran-4-one (868368-30-3)	Hydroxypyrone-based and Aryl backbone	Inhibits several MMPs	[126]
No. 582311-81-7	Phosphorus-based inhibitors and/or carbamoyl phosphonate zinc binding groups	Selectively inhibits MMP-1, 2, 3 8, 9, 14 and 13	[127,128,129]
Doxycycline	Tetracycline-based	Selectively inhibits MMP-2 and 9	[104,130]

## 7. Endogenous MMP Inhibitors

In addition to synthetic inhibitors, there is a class of endogenous inhibitors of MMPs of which the Tissue Inhibitors of Metalloproteinases (TIMPs) and α2-macroglobulin are the most represented [131]. α2-macroglobulin is a serum protein [132] capable of binding MMPs forming an inactive complex [133]. In a pioneering study, Kancha and co-workers have analyzed the protein levels of the α2-macroglobulin receptor (LRP/α2-MR). Its levels were found less expressed in the invasive sub-clones compared to the non-invasive ones derived from PC-3 and DU-145 human prostatic cells and the A2058 melanoma cell line supporting the hypothesis that the down-regulation of LRP/α2-MR complex can increase tumor cell invasiveness [134].

The TIMP inhibitors are grouped in a family composed of four different TIMPs, which are able to block the proteolytic action of the MMPs [135,136]. These inhibitors are capable of inhibiting MMPs binding their catalytic sites [131]. The TIMP molecules have 12 cysteine residues that form six loops through disulfide bonds, a conformation necessary to carry out the inhibitory activity towards MMPs. Indeed, the N-terminal site of TIMPs is able to bind the majority of MMPs, while the C-terminal site of TIMP-1 and TIMP-2 bind respectively to the hemopexin domain of pro-MMP-2 and pro-MMP-9 [137].

Normally, TIMPs can interact with different MMPs, however, MMP-1 is not targetable by TIMP-1 [133]. Nonetheless, inhibitory preferences exist; an example is represented by TIMP-1 that favors the inhibition of MMP-9 [138]. Besides its inhibitory action, TIMP-1 has also shown to have the ability to promote cell proliferation and the inhibition of apoptosis in a large number of cell types [139]. Normally, TIMPs levels are in excess compared to MMPs levels in extracellular fluids and tissues, thus limiting their activity to focal peri-cellular sites. As previously mentioned, TIMPs are known for their inhibitory activity towards MMPs, nonetheless, it has been shown that the activation of MMP-2 is associated with a low concentration of TIMP-2 coupled with MMP-14 in the cell membrane [12].

Changes in TIMPs levels are supposed to be involved in pathological conditions associated with unbalanced MMPs activities. [138]. For example, studies have suggested that an imbalance in the MMP-9/TIMP-1 ratio could be involved in the pathogenesis of COPD (chronic obstructive pulmonary disease) [140], but conflicting results were obtained from other studies [141,142]. Some evidence also suggests the role of TIMPs in cancer. In particular, alterations in TIMP-1 and MMP-9 levels have been observed in subjects with lung cancer compared to controls, associated with a poor prognosis, indicating a possible role of TIMP-1 and MMP-9 as a prognostic marker [143]. Other alterations in MMP-9 and TIMP-1 levels have been observed in subjects with breast cancer compared to controls. These dysregulations were associated with lymph node metastases and lower overall survival rates, suggesting that the levels of MMP-9 and TIMP-1 could be further evaluated to predict prognosis and progression of breast cancer [144].

TIMPs could be used as therapeutic indicators even in cardiovascular diseases, for instance for the treatment of atherosclerosis and aneurysm formation where elevated levels of MMP-9 were found [145]. As previously mentioned, each TIMP is able to inhibit several MMPs; therefore, it would be appropriate to develop engineered TIMPs able to interact with specific MMPs that play a role in that pathological process [133].

Hofmann and colleagues (2000) have made a review showing why the balance in the concentrations of MMPs and TIMPs is important; their dysregulation can determine the progression of melanoma. In particular, it has been demonstrated that the overexpression of TIMPs using recombinant TIMPs is able to reduce the invasiveness of melanoma cells [146].

Other studies have shown the importance of TIMP-1 in the control of melanoma progression. In particular, Khokha has showed that the B16-F10 cells previously transfected with the over-expressing construct for TIMP-1 and subsequently injected in mice, shown inhibition of the metastatic potential and growth compared to the non-transfected counterpart [147].

## 8. MMP-9 and Osteopontin

Osteopontin, indicated also as OPN or SPP1, is a sialylated protein of 300 aa belonging to the family of SIBLING (Small Integrin-Binding Ligand, N-linked Glycoprotein) proteins. It is codified by the *SPP1* gene (Secreted Phosphoprotein 1) located on the long arm of chromosome 4 region 22 (4q22.1) [148].

OPN was first isolated from bone tissue; then other studies demonstrated the presence of this protein in several tissues, e.g., epithelia, kidney, internal mucosae, carotid tissue [149,150,151].

OPN is involved in several functions, however, its major role is in bone remodeling. In particular, OPN is very important for bone replacement and resorption [152], because it is involved in the maturation of osteoclast and in the recruitment of osteoblast [153].

In addition, OPN is involved in immune processes, acting as an immunomodulator in several ways. It has been proved its involvement in chemotaxis, encouraging the migration of neutrophils in vitro and recruiting them into alcoholic liver disease [154,155]. Other functions, such as cytokine production, cell activation, and cell survival are mediated by OPN [156].

OPN is further involved in the block of apoptotic processes. Several harmful stimuli can induce apoptosis in both epithelial (fibroblasts and endothelial cells) and immune cells (macrophages and T-cells), but OPN is able to block these processes [157,158]. Several studies have been carried out to support the hypothesis that OPN improves the T cells survival [159]. These anti-apoptotic properties can lead to the survival of tumor cells [160]. This notion is supported by the fact that OPN is over-expressed in numerous tumors [161].

**Figure 3:**
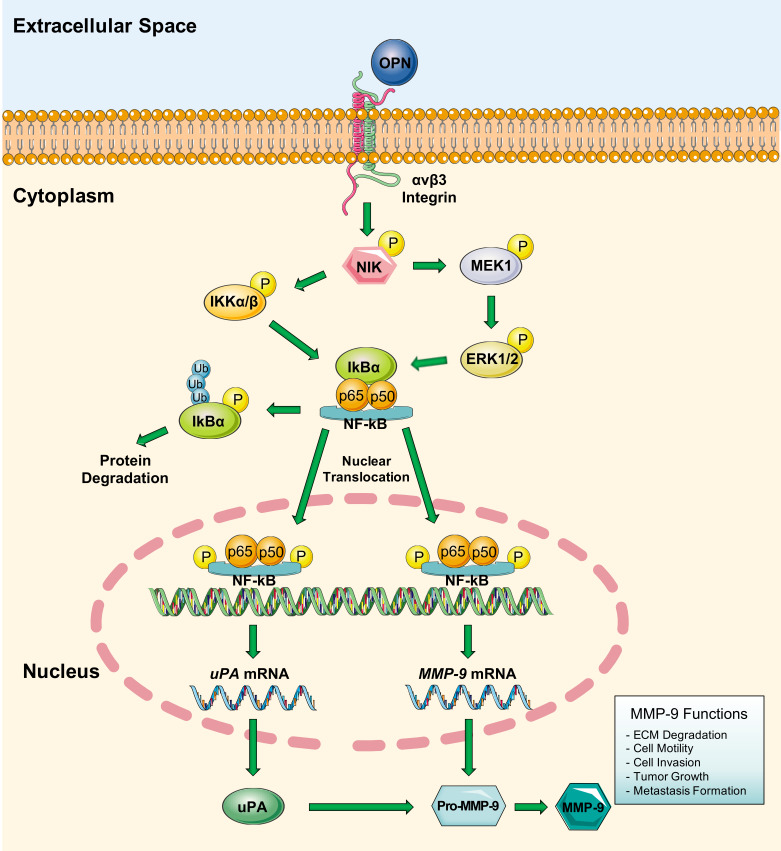
Schematic representation of MMP-9 activation mediated by OPN. The binding between OPN and αvβ3 integrin induce the phosphorylation and activation of NIK protein. NIK is able to induce the activation of the protein complex NF-κB/p65/p50 through two different mechanisms: (1) NIK activates IKKα/β then IKKα/β phosphorylated is able to disrupt the binding between NF-κB/p65/p50 complex and IkBα, thus, activating NF-κB and inducing IkBα ubiquitination and degradation; (2) NIK is able to activate MEK1/ERK1/2 cascade through phosphorylation, phosphorylated ERK1/2 thus activate NF-κB by removing IkBα. Finally, NF-κB/p65/p50 activated complex moves within the nucleus inducing the transcription and production of Pro-MMP-9 and uPA. This latter protein is able to bind Pro-MMP-9 leading to the production of MMP-9 active form.

With respect to the role of OPN in cancer, several studies have shown its ability to stimulate neo-angiogenesis [162] and to promote the development of metastases [163]. Its expression in tumors is a negative prognostic factor and it is generally associated with an increase in tumor proliferation and invasiveness supported also by the OPN-induced MMP-9 over-expression. Studies have shown that OPN and MMP-9 interact indirectly through the MAPK and the IKK/IkB/NF-κB pathways [34,164,165]. In particular, the link between OPN and αvβ3 integrin promotes the phosphorylated active form of NIK that in turn binds IKKα/β that activates NF-κB through the degradation of IκBα. NIK, when is coupled with OPN, induces also the activation of the MAPKs pathway responsible for the further activation of NF-κB and its accessory proteins p65 and p50. Consequently, OPN, stimulating the protein complex formed by NIK and IKK/MAPK, is able to up-regulate *MMP-9* transcription leading to the production of Pro-MMP-9. Finally, activated NF-κB stimulates also the transcription of *uPA* that in turn binds Pro-MMP-9 thus producing MMP-9 active form (Figure 3) [166].

The activation of MMP-9 mediated by OPN induces ECM degradation, cell invasion, tumor growth and metastasis. For example, the up-regulation of OPN, induced by (HIF-1)α and the activation of MMP-9, promotes gastric tumor metastasis. Finally, OPN is able to induce the activation of MAP3K1 signaling pathways regulated by MMP-9 resulting in the proliferation of melanoma cells and pulmonary metastases [167].

Youwen Zhou and co-workers have identified higher levels of OPN in melanoma cells than in normal nevi. To highlight the stimulatory effect of OPN on tumor progression, the KZ-28 melanoma cells were transfected with various concentrations of synthetic OPN small interference RNA (siRNA). Subsequently, cell viability assays have shown a decrease in the number of cells in a dose-dependent manner according to the increase of siRNA concentration. This is consistent with the stimulatory function of OPN on tumor cells [168].

On these bases, OPN and MMP-9 could be considered biomarkers of tumor progression [34].

## 9. Conclusions

The role of the MMP family and OPN have been widely described in order to understand their involvement in tumorigenesis, but the actual use of these proteins as biomarkers or therapeutic targets is still far away from happening. The paragraphs discussed above indicate that there is the need for discovering non-invasive biomarkers to assess the progression of melanoma both, during and after the therapy with small molecules, like Dabrafenib or other MAPK inhibitors. It has been also described in the use of MMPi drugs, with exciting preliminary results in vitro and in animal models, however, the expectation has declined rapidly because of the severe side effects observed in many clinical trials. Generally, the incongruences between the preclinical models and the clinical studies are probably due to the fact that in the preclinical models the drugs are administrated during the early phases of the disease when the conditions for severe and rapid worsening of tumor do not yet exist; while in humans, studies have been performed on patients with advanced tumors. So, it can be hypothesized that inhibition of MMPs could be effective in limiting tumor progression during its initial phase, keeping in mind that the efficacy of the therapy with MMPi drastically decreases with the progression of the disease.

On the other hand, the use of TIMPs, the endogenous inhibitors of several MMPs, has been suggested. An engineered TIMPs could be able to interact with a specific MMPs, and this is the reason why it has been hypothesized the use of these molecules to stop the tumor growth and migration.

Finally, the use of OPN and MMPs as biomarkers of progression in melanoma seems promising. Indeed, our recent evidence [32,33,34] showed that the OPN and MMP-9 are strongly involved in several neoplastic processes in melanoma, including ECM degradation, invasion of the surrounding tissues, metastasis formation and loss of apoptosis. Therefore, the evaluation of MMPs circulating levels, and in particular the use of OPN and MMP-9 as circulating biomarkers, may improve the current diagnostic strategies for melanoma and predict the aggressiveness of tumors, in order to personalize the therapeutic approach for the patients.

In this context, ongoing studies are attempting to screen melanoma patients for these biomarkers and further studies are needed to evaluate the predictive values of MMPs, TIMPs, OPN.

## Figures and Tables

**Table 1:** Classification of the MMP family members.

Sub-Family	Gene	Name	Chr Position	Cellular Position	Main Features [36,37]	Refs
Collagenases	*MMP-1*	Matrix Metallopeptidase 1 or Interstitial collagenase	Chr 11 q22.2	Extracellular space	ECM Substrates: Types I, II, and III, VII and X collagens. Function: Cleaves HIV Tat protein decreasing Tat’s mediated neurotoxicity in HIV infections.	[39,40]
	*MMP-8*	Matrix Metallopeptidase 8 or Neutrophil collagenase	Chr 11 q22.2	Extracellular space	ECM Substrates: Fibrillar type I, II, and III collagens.	[12]
	*MMP-13*	Matrix Metallopeptidase 13 or Collagenase 3	Chr 11 q22.2	Extracellular space	ECM Substrates: Fibrillar collagen, fibronectin, Aggrecan, Tenascin C, type I, III, IV, X, XIV collagens and especially soluble type II collagen. Functions: embryonic development (TGFB and CCN2 degradation), wound healing, bone development and mineralization, cartilage degradation, etc.	[41]
Gelatinases	*MMP-2*	Matrix Metallopeptidase 2, Gelatinase-A or 72 KDa Type IV Collagenase	Chr 16 q12.2	Extracellular space	ECM Substrates: Type I, II, III, IV, VII, X collagens and gelatin. Functions: ECM degradation, angiogenesis, tumor invasion, tissue repair, atherosclerotic plaque rupture, regulating of myocardial functions (via GSK3beta cleavage).	[42]
	*MMP-9*	Matrix Metallopeptidase 9, Gelatinase-B or 92 KDa Type IV Collagenase	Chr 20 q13.12	Extracellular space	ECM Substrates: Gelatin, fibronectin and type IV and V collagens. Functions: Angiogenesis, tumor invasion, tissue repair, tissue remodeling, etc.	[43]
Stromelysins	*MMP-3*	Matrix Metallopeptidase 3 or Stromelysin 1	Chr 11 q22.2	Extracellular space	ECM Substrates: Fibronectin, laminin, types I, III, IV, and V gelatins and type III, IV, X, and IX collagens. Functions: Degradation of cartilage proteoglycans and activation of procollagenase.	[44]
	*MMP-10*	Matrix Metallopeptidase 10 or Stromelysin 2	Chr 11 q22.2	Extracellular space	ECM Substrates: Fibronectin, elastin, type I, III, IV, and V gelatins. Functions: Activation of protocollagenasse and weak action toward type III, IV, and V collagens.	[44]
	*MMP-11*	Matrix Metallopeptidase 11 or Stromelysin 3	Chr 22 q11.23	Extracellular space	ECM Substrates: Type IV collagen, fibronectin, laminin and aggrecan. Function: Epithelial tumor invasiveness.	[45]
Matrilysins	*MMP-7*	Matrix Metallopeptidase 7 or Matrilysin	Chr 11 q22.2	Extracellular space	ECM Substrates: Casein, types I, III, IV, and V gelatins and fibronectin. Function: Activation of procollagenase.	[46]
	*MMP-26*	Matrix Metallopeptidase 26 or Matrilysin-2	Chr 11p15.4	Extracellular space	ECM Substrates: Fibronectin, fibrinogen, beta-casein, type IV collagen, type I gelatin and alpha-1 proteinase inhibitor. Function: Activation of progelatinase B.	[47]
Metalloelastase	*MMP-12*	Matrix Metallopeptidase 12 or Macrophage metalloelastase	Chr 11 q22.2	Extracellular space	ECM Substrates: Soluble and unsoluble elastin, type IV collagen and fibronectin. Function: Tissue remodeling after injuries.	[48]
Membrane-type MMPs	*MMP-14*	Matrix Metallopeptidase 14 or Membrane-Type-1 Matrix Metalloproteinase	Chr 14 q11.2	Plasma membrane	ECM Substrates: Gelatin, fibronectin, laminin and collagen. Functions: Activation of progelatinase A, skeletal and connective tissue development and remodeling, cell migration via pro-MMP-2 and MMP-15 binding.	[49]
	*MMP-15*	Matrix Metallopeptidase 15 or Membrane-Type-2 Matrix Metalloproteinase	Chr 16 q21	Plasma membrane	ECM Substrates: Gelatin, fibronectin, laminin and collagen. Function: Activation of progelatinase A.	[50]
	*MMP-16*	Matrix Metallopeptidase 16 or Membrane-Type-3 Matrix Metalloproteinase	Chr 8 q21.3	Plasma membrane	ECM Substrates: Several types of collagens and fibronectin. Functions: Activation of progelatinase A, angiogenesis and degradation and invasion of type I collagen by melanoma cells.	[44]
	*MMP-17*	Matrix Metallopeptidase 17 or Membrane-Type-4 Matrix Metalloproteinase	Chr 12 q24.33	Plasma membrane	ECM Substrates: Several ECM components, fibrinogen and fibrin. Functions: Binding of growth factors and cytokines during inflammation, tumor progression via pro-TNF-alpha cleavage.	[44]
	*MMP-24*	Matrix Metallopeptidase 24 or Membrane-Type-5 Matrix Metalloproteinase	Chr 20 q11.22	Plasma membrane	ECM Substrates: Proteoglycanase, fibronectin, N-cadherin. Functions: Induce degradation of dermatan sulfate and chondroitin sulfate proteoglycans, neurodevelopment and activation of progelatinase A.	[50]
	*MMP-25*	Matrix Metallopeptidase 25 or Membrane-Type-6 Matrix Metalloproteinase	Chr 10 q26.2	Extracellular space/Plasma membrane	ECM Substrates: Unknown Function: It may activate progelatinase.	[51]
Other MMPs	*MMP-19*	Matrix Metallopeptidase 19, Matrix Metallopeptidase RASI-1 or Matrix Metallopeptidase 18	Chr 12 q13.2	Extracellular space	ECM Substrates: collagen type IV, laminin, nidogen, nascin-C isoform, fibronectin, type I gelatin and aggrecan. Function: Cartilage matrix degradation in physio-pathological conditions.	[52]
	*MMP-20*	Matrix Metallopeptidase 20 or Enamelysin	Chr 11 q22.2	Extracellular space	ECM Substrates: Amelogenin, aggrecan and cartilage oligomeric matrix protein (COMP). Functions: Unknown	[53]
	*MMP-21*	Matrix Metallopeptidase 21	Chr 10 q26.2	Extracellular space	ECM Substrates: Alpha-1-antitrypsin Function: Embryogenesis vis NOTCH-signaling pathway inhibition.	[54]
	*MMP-23A*	Matrix Metallopeptidase 23A or Femalysin	Chr 1 p36.33	–	ECM Substrates: Unknown. Functions: Unknown.	[55]
	*MMP-23B*	Matrix Metallopeptidase 23B or Femalysin	Chr 1 p36.33	Extracellular space	ECM Substrates: Unknown. Functions: Unknown.	[55]
	*MMP-27*	Matrix Metallopeptidase 27	Chr 11 q22.2	Endoplasmic reticulum	ECM Substrates: Fibronectin, laminin, gelatins and/or collagens. Functions: Unknown.	[56]
	*MMP-28*	Matrix Metallopeptidase 28 or Epilysin	Chr 17 q12	Extracellular space	Substrates: Casein. Functions: Tissues homeostasis and repair.	[57]
